# Design and advanced characterization of quercetin-loaded nano-liposomes prepared by high-pressure homogenization

**DOI:** 10.1016/j.foodchem.2023.136680

**Published:** 2023-12-01

**Authors:** Sofia Melchior, Marta Codrich, Andrea Gorassini, Dora Mehn, Jessica Ponti, Giancarlo Verardo, Gianluca Tell, Luigi Calzolai, Sonia Calligaris

**Affiliations:** a Department of Agricultural, Food, Environmental and Animal Sciences, University of Udine, Udine, Italy; b Department of Medicine, University of Udine, Udine, Italy; c Department of Humanities and Cultural Heritage, University of Udine, Udine, Italy; d European Commission, Joint Research Centre (JRC), Ispra, Italy

**Keywords:** Liposomes, High-pressure homogenization, Advanced analytical techniques, Nano-sized delivery system

## Abstract

Quercetin-loaded nano-liposomes were prepared by high-pressure homogenization (HPH) at different pressures (up to 150 MPa) and number of passes (up to 3) to define the best processing conditions allowing the lowest particle size and the highest encapsulation efficiency (EE). The process at 150 MPa for 1 pass was the best, producing quercetin-loaded liposomes with the lowest particle size and 42% EE. Advanced techniques (multi-detector asymmetrical-flow field flow fractionation and analytical ultracentrifugation combined with transmission electron microscopy) were further used for the characterization of the liposomes which were oblong in shape (ca. 30 nm). Results highlight the need for several techniques to study nano-sized, polydisperse samples. The potential of quercetin-loaded liposomes against colon cancer cells was demonstrated. Results prove that HPH is an efficient and sustainable method for liposome preparation and highlight the remarkable role of process optimisation as well as the powerfulness of advanced methodologies for the characterisation of nano-structures.

## Highlights

• 150 MPa high-pressure homogenization produced nano-sized quercetin-loaded liposomes.
• Liposomes were oblong in shape (ca. 30 nm) and had a 42% encapsulation efficiency.
• Advanced analytical techniques are required to characterize nano-sized liposomes.
• Loaded liposomes exhibited higher antitumor action compared to free quercetin.

## Graphical abstract

**Figure:**
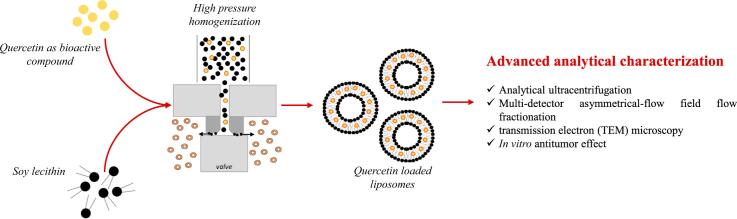

## 1 Introduction

Liposomes are self-assembled vesicles having dimensions in the nanometre to micrometre range characterized by a phospholipid bilayer structure separating the internal from the surrounding aqueous environment (Liu et al., 2020). Due to their unique properties, such as biocompatibility, target release, and the possibility to deliver both water-soluble and insoluble compounds, liposomes are well-recognised as smart delivery systems for pharmaceutical, agricultural, cosmetic and, more recently, food applications (Barroso et al., 2021, Liu et al., 2020). The effectiveness of liposomes as delivery systems greatly depends on their dimensions, which affect not only their behaviour in food products during processing and storage, but also their physiological fate in terms of adsorption, biodistribution in the bloodstream, and excretion (Gioria et al., 2018, Haddadzadegan et al., 2022). The smaller the particle size, the greater the specific surface area exposed to lipase digestion, which may influence the release of the encapsulated bioactive compound and thus its bioaccessibility (Álvarez et al., 2022). Additionally, the smaller the size, the higher the diffusion coefficient in intestinal mucus especially when the nanocarrier has dimensions below 100–200 nm, being in the mesh size of the mucus microstructure (Haddadzadegan et al., 2022).

In this regard, attention should be paid to the methodology applied to study the morphology and particle dimensions of liposomes. It is well-recognised that a robust nanometric assessment can be obtained only by using a combination of analytical techniques (Calzolai, Gilliland, & Rossi, 2012). Traditionally, dynamic light scattering (DLS) in batch mode is applied to gain information about the particle size distribution of liposomes. However, this technique is considered to be a low-resolution method due to the strong dependence of the scattering intensity on the particle size. DLS might generate misleading results in complex media containing multiple populations with similar sizes (Caputo et al., 2019a, Caputo et al., 2019b, Gioria et al., 2018, Mehn et al., 2017a). Other analytical methods have been proposed for the deep characterization of liposomes. Among them, multi-detector asymmetrical-flow field flow fractionation (AF4) and analytical ultracentrifugation (AUC) are particularly valuable to obtain a more realistic evaluation of particle size and polydispersity. AUC monitors the sedimentation profile of particles in real-time and, by the application of a suitable mathematical model, allows the calculation of the particle diameter in complex matrices (Planken & Cölfen, 2010). AF4 is a high-resolution sizing technique thanks to the introduction of a fractionation step separating particles based on their size, without affecting their “soft” structure. Moreover, AF4 offers the possibility to be combined with multiple online detectors, overcoming the limitations of traditional batch mode DLS (Caputo et al., 2019a, Parot et al., 2020). To our knowledge, AUC and AF4 have been scarcely applied to study liposomes for food applications, even if these techniques are widely used in the pharmaceutical sector for the characterization of nano-sized liposomes (Iavicoli et al., 2015, Mehn et al., 2017b, Mehn et al., 2020) and lipid nanoparticles (Caputo et al., 2019a).

It is well known that the formulation and preparation methodologies have a pivotal impact on controlling particle size, shape, polydispersity, and finally functionality of liposomes. Traditionally, reverse-phase evaporation, thin film rehydration, and freeze–thaw cycling are the most widely used methods to form liposomes intended as delivery systems (Jahn et al., 2007, Wang et al., 2017). In all cases, these manufacturing methodologies require the use of organic solvents and are not suitable for massive production (Wang, Chen, Guo, & Huang, 2023).

Considering food applications, additional restrictions may be considered. Ingredients must be GRAS (Generally Recognised As Safe) and available at a convenient cost. Moreover, the manufacturing method must avoid the use of solvents and be easy-scalable with low-cost and low-energy consumption. In this context, high-pressure homogenization (HPH), being a continuous, cheap, easy to scale-up and eco-friendly process, appears promising (Donsì et al., 2013, Floury et al., 2004). During the HPH process, a fluid is pumped through a narrow gap valve of a pressure intensifier and exposed to intense mechanical forces and elongational stress at the valve entrance and in the valve gap while turbulence, cavitation, and impacts of the solid surface occur at the gap outlet (Floury et al., 2004). Industrial-scale high-pressure homogenizers are already available and used for manifold applications by the food industry (*e.g.* emulsification, microbial load reduction, biopolymer structuring, and cell disruption) (Mesa et al., 2020). It has been previously demonstrated that HPH could be able to generate nano-sized liposomes, without the use of organic solvents and with good encapsulation efficiency (Ibišević et al., 2019, Wagner and Vorauer-Uhl, 2011). Guldiken, Gibis, Boyacioglu, Capanoglu, and Weiss (2017) demonstrated the efficiency of HPH (155 MPa for 3 passes) for the production of nano-sized liposomes (around 45 nm) vehiculating black carrots anthocyanins; while Wang et al. (2017) used HPH at 70 MPa for 3 passes for the preparation of liposomes of 120 nm size delivering phytosterols. Similar results were obtained when polyphenol-rich grape seed extract was incorporated into liposomes after 5 passes at 155 MPa (Gibis, Rahn, & Weiss, 2013). Considering literature results and to the best of our knowledge, it is quite difficult to find data on the impact of HPH processing conditions (*i.e.,* pressure and the number of passes) on the characteristics and encapsulation efficiency of liposomes. In general, it is expected that a progressive decrease in liposome size could occur along with increasing process intensity in terms of pressure and number of passes (Barnadas-Rodríguez & Sabés, 2001). However, no clear information is reported about this correlation in the literature.

Based on these considerations, the present study aimed at preparing and characterizing nano-liposomes designed for the delivery of quercetin by using HPH. Quercetin is a flavonoid with a well-accepted health-promoting capacity, due to antioxidant, anticarcinogenic and anti-senescence actions (Mária & Ingrid, 2017). Interestingly, quercetin is also an antitumor agent inhibiting cell growth of different types of cancer (Rauf et al., 2018). In particular, quercetin inhibits cell viability of colon cancer cells inducing apoptosis and causing G2 arrest (Kee et al., 2016, Zhang et al., 2015).

Quercetin is poorly absorbed by the digestive system, especially in the stomach. Liposomes could allow the delivery of quercetin in the bowel which offers a more prone environment for absorption, thanks to its intrinsic characteristics, including less digestive enzymatic activity and a larger surface area contribution in the human digestive tract (Singhal, Jain, Singhal, Elias, & Showkat, 2011).

This study was first dedicated to the optimization of the HPH process allowing the formation of quercetin-loaded liposomes made by soy-lecithin. To this purpose, HPH treatments at different homogenization pressures (up to 150 MPa) and number of passes (up to 3) were applied. Then, liposomes obtained by using the best processing conditions were further characterized by AF4 and AUC in combination with transmission electron microscopy (TEM). Finally, the toxic effect of quercetin-loaded structures against colon cancer cells was evaluated using a metabolic *in vitro* assay to prove the potential biological effect of the obtained liposomes. The results presented in this work show the remarkable role of process optimization and the powerfulness of advanced techniques for the characterization of liposomes.

## 2 Materials and methods

### 2.1 Materials

Soy lecithin was purchased from Carlo Erba Reagents SpA (Milan, Italy); potassium phosphate (K_2_HPO_4_), quercetin (≥95%), kaempferol (≥97%; HPLC-DAD Internal Standard; IS), dimethyl sulfoxide (DMSO), methanol (MeOH), and formic acid for HPLC-MS were purchased from Sigma-Aldrich (Milan, Italy). HCT-116 p53^+/+^ cells were provided by ATCC (Manassas, USA). Dulbecco’s modified Eagle’s medium, foetal bovine serum, GlutaMAX, penicillin, *N*-GARDE Mycoplasma PCR Reagent set, and streptomycin were provided by EuroClone (Milan, Italy). The copper Lacey Carbon Film grid was purchased from Agar Scientific (Stansted, UK). Milli-Q grade water was produced by the Elgastat UHQ-PS system (ELGA, High Wycombe, Buckinghamshire, UK).

### 2.2 Liposome preparation

Soy lecithin (10 g/L) with or without quercetin (0.3 g/L) was dispersed in water and stirred for 1 h at room temperature. The dispersion was then pre-homogenized with a high-speed homogenizer (Ultra-Turrax Homogenizer, IKA, Staufen, Germany) for 5 min at 14,000 rpm. Then, the sample was homogenized using a continuous lab-scale high-pressure homogenizer (Panda Plus 2000; GEA Niro Soavi, Parma, Italy) equipped with two Re + type tungsten carbide homogenization valves and a flow rate of 10 L/h. The treatment was conducted at pressures of 50, 100, and 150 MPa for 1 pass and at 150 MPa for 3 passes. The temperature was maintained below 40 °C. A sample not subject to HPH treatment was used as a control (No HPH). To remove unincorporated material, after HPH, samples were centrifuged (AvantiTM centrifuge, J-25, Beckman Coulter, Brea, CA, USA) for 10 min at 10,000× *g* and the supernatant containing liposomes was collected for further analysis, while the precipitate was discarded. Samples were prepared with and without quercetin and stored at 4 °C until further analysis.

### 2.3 Analytical determinations

#### 2.3.1 Turbidity measurement

Turbidity was measured at 660 nm using a UV–Vis spectrophotometer (UV-2501 PC, Shimadzu, Kyoto, Japan) at 25 °C with a 1 cm path-length cuvette.

#### 2.3.2 Particle size distribution and ζ-potential

Particle size distribution in batch mode was measured by dynamic light scattering (DLS, Zetasizer NanoZS, Malvern Instruments, Worcestershire, UK). Samples were diluted 1:100 (v/v) with MilliQ water and placed in a cuvette in which the scattered laser light (at 173° angle) was analysed.

For ζ-potential measurements, samples were placed in a disposable folded capillary cell equipped with two electrodes to assess particle electrophoretic mobility.

#### 2.3.3 Encapsulation efficiency by HPLC-ESI-MS-DAD

Encapsulation efficiency (EE, %) was measured by HPLC-ESI-MS-DAD. Samples were diluted 1:20 (v/v) with methanol to allow the release of quercetin from the liposomes.

Each sample (100 μL) was spiked with 100 μL of the IS solution (kaempferol, 9.7 μg/mL in MeOH) and diluted in 1 mL of MeOH/H_2_O 70:30 (v/v) with 0.4% of formic acid. The sample was transferred in an autosampler vial for the HPLC-ESI-MS-DAD analysis.

Chromatographic analysis was performed with a UHPLC Ultimate 3000 (Thermo Scientific, San Jose, CA, USA) equipped with a column oven and a thermostated autosampler. The chromatographic separation was performed using a column InfinityLab Poroshell 120 EC-C18 (4.6×150 mm, 2.7 μm; (Agilent Technology, Milan, Italy), thermostated at 30 °C. Elution was carried out at a flow rate of 0.3 mL/min, using as mobile phase a mixture of 0.2% formic acid in methanol (A) and 0.2% formic acid in water (B) with the following gradient: 0–13 min 70% A, 15 min 100% A, 26 min 100% A, 28 min 70% A, 35 min 70% A. The injection volume was 20 μL. The HPLC system was coupled with an Ultimate 3000 RS diode array detector (DAD; Thermo Scientific, San Jose, CA, USA) and a Finnigan LXQ linear ion trap mass spectrometer with an electrospray ionization source (ESI-MS; Thermo Scientific, San Jose, CA, USA), in parallel by splitting the mobile phase 1:1. The acquisition was carried out in full scan (*m*/*z* 50–1500) and in full scan MS^2^ (*m*/*z* 50–600) selecting the precursor ion [M – H]^–^ at *m*/*z* 301.1 for quercetin and at *m*/*z* 285.2 for kaempferol (IS). The quantitative analysis was carried out using a diode array detector controlled by Chromeleon software (version 6.80). Spectral data from all peaks were accumulated in the range of 200–400 nm and chromatograms were recorded at 372 nm for both quercetin and kaempferol (IS).

A stock solution of quercetin in MeOH/H_2_O 70:30 (v/v) with 0.4% of formic acid was serially diluted with the same solvent, with a constant concentration of kaempferol (0.97 μg/mL), to prepare 6-point calibration curve in the range 0.1–3.0 μg/mL. The R^2^ coefficient for the calibration curve was >0.999.

EE (%) was calculated following Eq. (1):

(1) $$EE\left(\%\right)=\frac{\mathrm{encapsulated}\;\mathrm{quercetin}(\frac{\mathrm{mg}}{\mathrm{mL}})}{\mathrm{quercetin}\;\mathrm{in}\;\mathrm{the}\;\mathrm{initial}\;\mathrm{dispersion}(\frac{\mathrm{mg}}{\mathrm{mL}})}x100$$

#### 2.3.4 Analytical ultracentrifugation (AUC)

AUC analysis was performed using a Beckman Coulter ProteomeLabTM XL-I analytical ultracentrifuge (Brea, CA, USA) equipped with an 8-hole rotor, collecting interference and absorbance signals simultaneously in the same run. Samples were diluted with water and measurements were performed at 20 °C at a rotation speed up to 30,000 rpm. Absorbance signals were collected at 250 and 370 nm. The ls-g*(s) distribution model of the SEDFIT software was applied to fit experimental data to calculate the sedimentation coefficient distribution. The latter was transformed to hydrodynamic diameter (Dh) using the “transform s distribution to r distribution” option of SEDFIT. Particle viscosity and density were calculated in accordance with the ISO standard n. 18747–2 (ISO, 2019). Samples were diluted in sucrose solutions (5% w/v) and the reference cell of the AUC centrepiece was loaded with sucrose solution of the same density.

#### 2.3.5 Multi-detector asymmetrical-flow field flow fractionation (MD-AF4)

The analysis was performed with AF2000 Multiflow FFF (Postnova Analytics GmbH, Landsberg, Germany) equipped with isocratic pumps (PN1130, Postnova Analytics GmbH, Landsberg, Germany), degasser (PN7520, Postnova Analytics GmbH, Landsberg, Germany), and a manual injector with a 50 µL loop. The system was equipped with three online detectors: multi-angle laser scattering (MALS, DAWN Wyatt, Santa Barbara, CA, USA) detector (λ = 661 nm) equipped with 18 detectors at angles from 12.8° to 157.8°; UV–VIS detector (3212, Postnova Analytics GmbH, Landsberg, Germany) set at 250 nm; and DLS (Zetasizer NanoZS, Malvern Instruments, Worcestershire, UK) equipped with flow-mode cell, detecting in backscattering at 173°. The mobile phase, consisting of phosphate buffer 1 mM (pH 8), was pumped into the channel from opposite ends during injection and focusing and passed through a 10 kDa cellulose membrane (294 mm × 30 mm, Postnova Analytics GmbH, Landsberg, Germany) at the accumulation wall. When focusing was complete the analyte formed a thin line transverse to the direction of the channel and detector flow. Subsequently, the analyte was separated into components based on the size as a result of opposing forces of particle diffusion and applied cross-flow rate perpendicular to the channel flow (Parot et al., 2020). The optimized method consisted of a focus-injection step for 5 min, followed by elution at a detector flow of 0.5 mL/min with an exponentially decaying cross flow from 0.6 to 0 mL/min for 60 min. In this experiment, 50 µL aliquots of the sample were used. Online UV–Vis absorbance at 250 nm was utilized for the estimation of mass recovery (R%). The latter was obtained by integrating the area under the UV–Vis peak for each sample eluted with and without the applied cross flow and focusing step using Nova FFF4 software (Postnova Analytics GmbH, Landsberg, Germany) (Eq.2).

(2) $$R\left(\%\right)=\frac{UV-Vis\;area\;of\;fractionated\;sample}{UV-Vis\;area\;of\;sample\;without\;crossflow\;or\;focus}*100$$

Although different parameters were considered and modified (cross flow, injection time, sample amount and concentration, mobile phase composition, and pH) to optimize the process, the maximum recovery (R%) obtained in this study was below 70% which is the minimum limit required by ISO/TS 21,362 (ISO, 2018). However, the fractogram and the increase in particle size with the exit time indicated that the applied method was satisfactory to achieve the separation of liposomes having different sizes while guaranteeing high reproducibility (Iavicoli et al., 2015).

The geometric diameter (Dg) was computed through the Wyatt ASTRA software (Santa Barbara, CA, USA) by using the Zimm model, typically applied for small particles, and the sphere model obtaining R^2^ higher than 0.7 and 0.9, respectively. The hydrodynamic diameter (Dh) was obtained by analysing DLS flow data with the Zetasizer software (Malvern Instruments, Worcestershire, UK). The shape factor was calculated as the ratio between Dg and Dh. For each sample, the analysis was performed in triplicate.

#### 2.3.6 Transmission electron microscope (TEM)

The shape and the size of liposomes were analysed by direct visualization using the Cryo-TEM technique (Burrows & Penn, 2013). Briefly, aliquots of 4 µL of liposomes stock suspension were deposited on 200 mesh copper Lacey Carbon Film grid pre-treated by Leica EM ACE200 glow discharge (10 mA, 30 s; Leica, Milan, Italy). Excess material was blotted away by filter paper and sample vitrification was done using Leica EM GP (Leica, Milan, Italy) equipment. The sample was immediately analysed by TEM at 120 kV (JEOL-JEM 2100, JEOL, Milan, Italy) equipped with Gatan cryo transfer holder 626 (Gatan, Pleasanton, CA, USA).

### 2.4 Cell cultures

HCT-116 p53^+/+^ (ATCC) cells were grown in Dulbecco’s modified Eagle’s medium supplemented with 10% foetal bovine serum, 2 mM GlutaMAX and 100 U/mL penicillin and 10 µg/mL streptomycin. The cells were tested for mycoplasma by PCR-based mycoplasma testing (*N*-GARDE Mycoplasma PCR Reagent set PCR kit for Mycoplasma Detection # EMK090020).

#### 2.4.1 Cell metabolic assay

Cell line viability was measured using the colorimetric method CellTiter 96® AQueous One Solution Proliferation assay (MTS) (Promega, Madison, WI, USA) according to the manufacturer’s protocol. MTS assay is a colorimetric method that measures the reduction of MTS tetrazolium compound into formazan in mitochondrial metabolically active cells. In detail, 5000 cells were plated on transparent 96-well plates. The day after, cells were treated with quercetin-loaded liposomes or quercetin alone at concentrations of 3.125, 6.25, 12.5, 25, 50, and 100 µM. The control cells were treated with unloaded liposomes at the same lecithin concentration of empty liposomes and the vehicle DMSO, respectively.

The metabolic activity of the viable cells was measured by recording the absorbance at 490 nm with a plate reader after 48 h (Enspire PerkinElmer, Waltham, MA, USA).

### 2.5 Statistical analysis

Results are averages of three technical replicates from three independent assays and are reported as means ± standard deviation. Analysis of variance (ANOVA) and *t*-test were performed by using R v. 4.1.1 for Windows (The R foundation for statistical computing, Vienna, Austria). A Tukey’s *post-hoc* test was used to assess differences between means (*p* < 0.05).

## 3 Results and discussion

### 3.1 Definition of the best processing condition for liposome preparation

The effect of high-pressure homogenization (HPH) conditions (pressure and number of passes) on liposome formation was evaluated. Table 1 shows turbidity, particle size distribution, ζ-potential, and encapsulation efficiency (EE) of liposomes with and without quercetin.

**Table 1:** Absorbance at 660 nm, ζ-potential, Z average (Z-ave), polydispersity index (PDI), and encapsulation efficiency (EE) of liposomes with and without quercetin produced by high-speed homogenization (control) and high-pressure homogenization (HPH) at different conditions.

Presence of quercetin	Homogenization pressure	Number of passes	Turbidity (-)	Z-ave (nm)	PDI (-)	ζ-potential (mV)	EE (%)
without quercetin	0 (control)	–	0.405±0.003 ^a,B^	163.8±2.4 ^a,B^	0.522±0.008 ^a,A^	−20.20 ± 0.79 ^a,B^	
	50 MPa	1	0.122±0.004^b,B^	96.1±1.5^b,B^	0.349±0.035^b,B^	−21.87 ± 1.11 ^bc,A^	
	100 MPa	1	0.055±0.001 ^d,B^	73.4±1.4^c,B^	0.292±0.019 ^cd,B^	−20.73 ± 0.68 ^ab,A^	
	150 MPa	1	0.047±0.001 ^e,B^	69.3±0.8 ^d,B^	0.282±0.007 ^d,B^	–23.72 ± 0.62 ^d,B^	
	150 MPa	3	0.062±0.001^c,A^	53.7±1.5 ^e,B^	0.331±0.035 ^bc,B^	–22.08 ± 0.45^c,B^	

with quercetin	0 (control)	–	0.720±0.001 ^a,A^	267.0±10.2 ^a,A^	0.529±0.018 ^a,A^	−18.50 ± 1.21 ^a,A^	32.93±0.12^b^
	50 MPa	1	0.282±0.062^b,A^	117.0±4.4^b,A^	0.437±0.027^b,A^	−21.70 ± 1.10^b,A^	37.04±0.13^ab^
	100 MPa	1	0.109±0.028^c,A^	88.4±6.3^c,A^	0.464±0.049^b,A^	−21.58 ± 1.33^b,A^	34.13±0.24^b^
	150 MPa	1	0.085±0.001^c,A^	92.6±1.2^c,A^	0.323±0.009^c,A^	−21.94 ± 1.39^b,A^	42.11±4.08^a^
	150 MPa	3	0.056±0.000^c,B^	72.0±2.9 ^d,A^	0.550±0.019 ^a,A^	−20.04 ± 0.81 ^ab,A^	31.19±0.06^b^

Mean ± standard deviation (n > 2). ^a−e^: means indicated a significant difference (*p* < 0.05) among different treatments; ^A−B^: means indicated a significant difference (*p* < 0.05) among loaded and unloaded liposomes.

The turbidity of unloaded liposomes decreased as the homogenization pressure increased up to 150 MPa. The further increase in the number of passes at 150 MPa led to turbidity rise. Since turbidity is related to the size and distribution of particles, particle size distribution was also analysed (Fig. S1). All samples were polydisperse as indicated by PDI values higher than 0.2. The control sample presented the highest Z-average (Z-ave) and PDI above 0.5 indicating a very broad size distribution (Fig. S1a). The application of HPH at increasing intensity induced the formation of liposomes with lower polydispersity. The PDI decreased as a function of pressure increase and as a result of increased shear forces suffered by the sample during the passage through the homogenization valve. The sample prepared at 150 MPa for 1 pass had the lowest PDI (0.282±0.007) and Z-ave (69.3±0.8 nm). These results were in agreement with previous studies that reported the dependence of particle size reduction on HPH pressure increase (Barnadas-Rodríguez & Sabés, 2001). Although Z-ave was the lowest, the PDI of liposomes significantly increased (*p* < 0.05) by applying 150 MPa for 3 passes. The reduction of size homogeneity can be associated with aggregation and/or destabilization of particles upon treatment (Ibišević et al., 2019).

Similar results were observed for quercetin-loaded liposomes. However, comparing unloaded and loaded samples, the presence of quercetin led to higher turbidity and larger particle dimensions (Fig. S1). This phenomenon was also observed by Toniazzo, Peres, Ramos, and Pinho (2017) who found that the average particle size of quercetin-loaded liposomes was higher than that of unloaded ones. Similarly, Gibis, Ruedt, and Weiss (2016) observed the same behaviour when grape-seed polyphenols were incorporated into liposomes. The increase in particle size of loaded structures may be attributed to the location of quercetin closer to the centre of the lipid bilayer. This may adversely affect the colloidal stability of liposomes by reducing the surface exposure of the phosphate head groups leading to system destabilization (Malekar, Sarode, Bach, & Worthen, 2016).

The ζ-potential (Table 1) becomes more negative by increasing the pressure up to 150 MPa, while a slight increase was observed for the sample produced at 150 MPa for 3 passes probably due to the system destabilization, in agreement with previous results. The progressive tendency of ζ-potential to get close to −30 mV indicated that the physical stability of liposomes increased with pressure (Madureira et al., 2015). Interestingly, the effect of the treatment was less pronounced in loaded liposomes, as a consequence of the quercetin embedded in the lecithin bilayer, which leads to reduced exposure of phosphate groups (Malekar et al., 2016), as previously speculated.

Finally, encapsulation efficiency (EE) (Table 1) ranged from 31% to 42%. These results are in agreement with the literature reporting EE between 32% and 50% in the case of liposomes containing black carrot extract produced by HPH (Guldiken et al., 2017). As expected, and in agreement with the previous observation, EE increased as a function of pressure increase, achieving the highest value at 150 MPa, while the lowest one was obtained at 150 MPa for 3 passes. Besides the process conditions, EE is expected to be affected by the solubility of quercetin in the lecithin bilayer (Guldiken et al., 2017). Complete liposomal encapsulation may be achieved only when the encapsulated material is soluble in the liposome membrane (Bryła, Lewandowicz, & Juzwa, 2015), as observed considering the delivery of hibiscus (61–72%), elderberry (25%), and grape seed (88%) (Bryła et al., 2015, Gibis et al., 2016, Gibis et al., 2014) extracts.

Based on the obtained results, the process at 150 MPa for 1 pass was considered the best-performing one allowing to obtain nano-sized liposomes with a good encapsulation efficiency. From an industrial perspective, these results could open interesting opportunities for scaling-up because only one pass is required to generate low-size liposomes, allowing the application of a continuous process. Additionally, it should be stressed the remarkable role of a process optimization step before scaling-up not only to avoid dangerous over-processing in terms of liposome structural integrity but also to reduce process costs and energy consumption.

### 3.2 Nanostructure of liposomes

Quercetin-loaded and unloaded liposomes, produced by applying the best performing condition (150 MPa for 1 pass), were in-depth characterized for their nanostructure by analytical ultracentrifugation (AUC) and asymmetric flow-field flow fractionation (AF4).

**Fig. 1:**
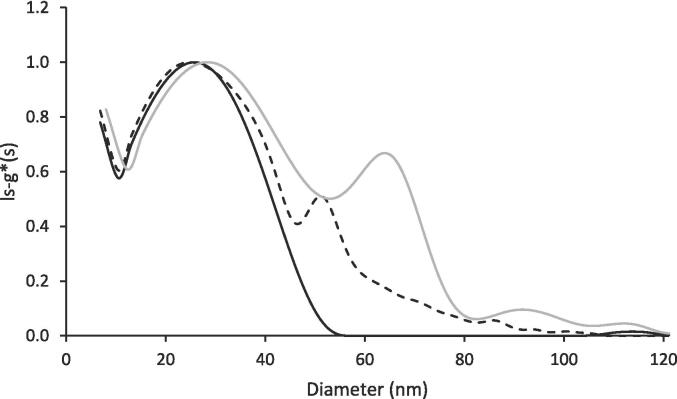
Size distribution measured with AUC of liposomes (black line) and liposomes with quercetin (dotted black line) calculated from the absorption-based measurement at 250 nm. The grey line indicates the distribution of loaded liposomes with the adsorption-based measurement at 370 nm.

Fig. 1 reports the size distribution of liposomes acquired at 250 nm. Unloaded liposomes were characterized by an almost monodisperse distribution with a mean diameter of around 30 nm. The same particle family was also detected in quercetin-loaded samples, but, in this case, the distribution was broader. An additional peak was observed at 52 nm, while larger particles were present to a lesser extent. In agreement with previous data, the addition of quercetin interfered with liposome formation resulting in structures with larger dimensions (Malekar et al., 2016). To have a better insight into the dimension of quercetin-loaded liposomes, samples were also analysed at 370 nm, at the absorbance maximum specific to quercetin. At this wavelength, the presence of liposomes with a size around 65 nm was observed.

AUC results highlighted that the size of the majority of particles was almost three times lower than that measured by DLS in batch mode, which was 99.3±4.5 nm and 110.6±2.5 nm for unloaded and loaded samples, respectively (Fig. S1d). Such findings highlighted the resolution limitations of DLS in batch mode, confirming the necessity to apply other techniques when dealing with particle size characterization at the nano-scale (Caputo et al., 2019a, Gioria et al., 2018).

**Fig. 2:**
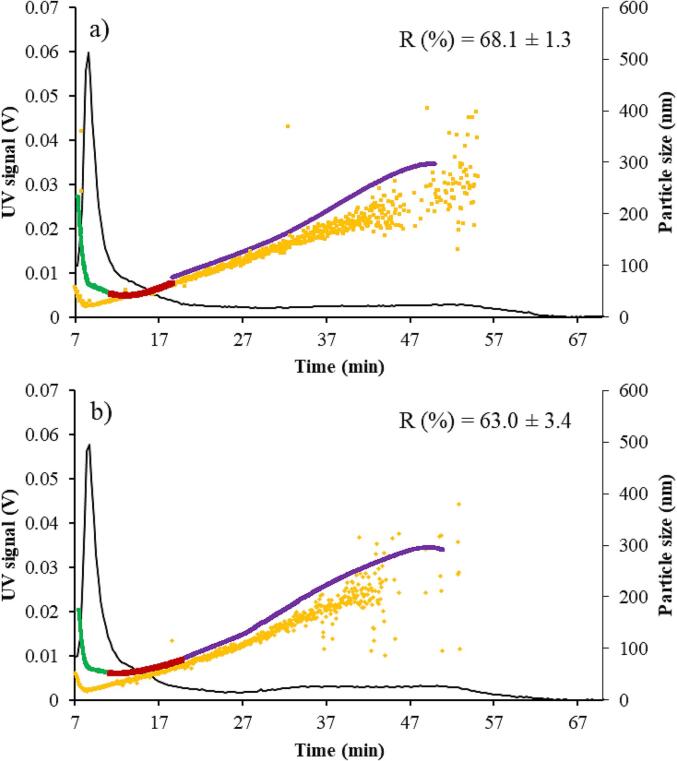
MD-AF4 fractograms of liposomes (a) and loaded liposomes with quercetin (b). Black line: UV–Vis linear response at 250 nm; yellow dots: hydrodynamic diameter obtained by DLS in flow mode; green and red dots: geometric diameter of the first two size populations detected by MALS and elaborated by the Zimm model; violet dots: geometric diameter of the third size population detected by MALS and calculated by the Sphere model. The maximum recovery for both loaded and unloaded liposomes is reported in the insight. (For interpretation of the references to colour in this figure legend, the reader is referred to the web version of this article.)

AUC results were supported also by data acquired by AF4 using different detectors (*i.e.,* UV–Vis, MALS, and DLS) (Fig. 2).

Observing the UV–Vis-based elugram of unloaded samples (black line in Fig. 2a), two main peaks were detected. The first one (8.6 min) showed the highest intensity, while the second one (between 25 and 60 min) was less intense and broader. To obtain information about the geometric diameter (Dg) of liposomes and their abundance, UV–Vis data were combined with those acquired by the MALS detector. Three main particle families having different dimensions were observed. Two of them (green and red lines in Fig. 2a) were detected at the highest UV–Vis intensity, indicating that the majority of liposomes belonged to these particle families with Dg ranging from 42 to 65 nm. The third particle family (violet line in Fig. 2a) appeared with the second UV–Vis peak, indicating the presence of liposomes having dimensions higher than 80 nm. Quercetin-loaded liposomes (Fig. 2b) showed the same behaviour but, in this case, the dimensions of the smallest particles were slightly higher (50–71 nm).

The availability of the online Dg obtained by MALS, as well as the hydrodynamic diameter (Dh) detected with DLS in flow mode (yellow dots in Fig. 2), allowed us to gain information on the morphology of particles by calculating the shape factor (ρ). It is interesting to note that the majority of liposomes, independently from the addition of quercetin, presented Dg higher than Dh, resulting in ρ > 1, which is typical of oblong nanoparticles (*i.e.* nanorods, nanotubes, or nanofibers) (Iavicoli et al., 2015). As observed for drug delivery, rod-shaped particles or non-spherical ones are taken up and transported across the intestinal cells to a greater extent compared to those with spherical shapes (Banerjee, Qi, Gogoi, Wong, & Mitragotri, 2016). Thus, the morphological heterogeneity of our samples could have a favourable effect on the potential biological activity of encapsulated quercetin (Zhou & McClements, 2022).

**Fig. 3:**
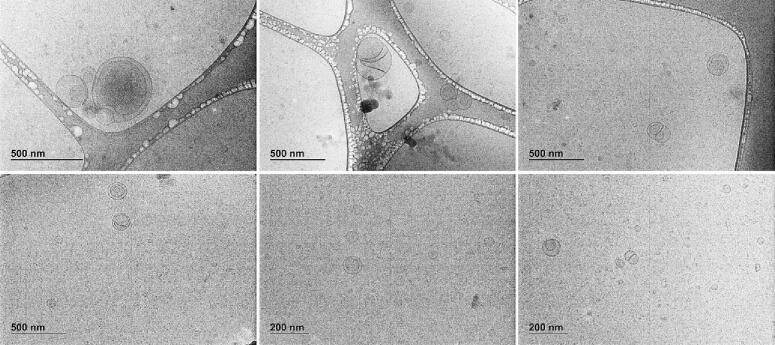
Cryo-TEM micrographs of liposomes.

Shape and size data were corroborated by direct visualization of liposomes with Cryo-TEM (Fig. 3). TEM micrographs highlighted the presence of structures with heterogeneous dimensions in the same range previously detected by AUC (Fig. 1) and AF4 (Fig. 2). Moreover, spherical and oblong liposomes were observed, confirming the previous hypothesis. In this regard, it is interesting to note that the HPH treatment applied was able to generate liposomes with different structures (*i.e.,* unilamellar, multilamellar, and multivesicular vesicles), improving the stability and delaying the sustained release of quercetin (Liu et al., 2020).

### 3.3 *In vitro* toxic effect on a cancer cell line

**Fig. 4:**
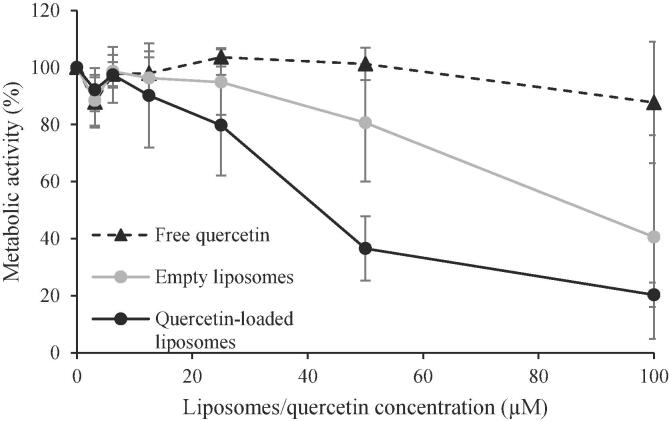
Quercetin-loaded liposomes affect cell metabolism in a colon cancer cells model. MTS assay on HCT-116 p53^+/+^ cells. Cells were treated with free quercetin (black dotted line), empty liposomes (gray line), or quercetin-loaded liposomes (black line) increasing concentrations for 48 h. In the graph, the percentage of metabolic activity relative to untreated cells (for empty liposomes and quercetin loaded liposome-treated cells) and to DMSO-treated cells (for free quercetin-treated cells), both arbitrarily set to 100%, is represented.

To determine the biological efficiency of quercetin-loaded liposomes and test their toxic effects, a colon cancer cell line (HCT-116 p53^+/+^ cells), that is wild-type for TP53 gene expression (Bunz et al., 1998), was employed. Experimentally, HCT-116 p53^+/+^ cells were treated with increasing concentrations of quercetin-loaded liposomes or free quercetin for 48 h. The metabolic activity of viable cells was evaluated by using the MTS assay (Fig. 4). Data obtained clearly showed that the HCT-116 p53^+/+^ cell line was significantly more sensitive to quercetin-loaded liposomes, at doses greater and equal to 50 μM, than to free quercetin (*p* < 0.05).

Notably, also unloaded liposomes had a slight effect on cancer cell metabolism. This result could be attributed to soy lecithin, which is involved in several metabolic and intracellular processes, such as the induction of necrosis and apoptosis. Previous studies have also demonstrated that surface active molecules (*i.e.* lecithin, bile salts, phospholipids as well as anionic, cationic, and nonionic surfactants) may reduce the viability of model colon cancer cells through several mechanisms, including the disruption of cell membranes, the modification of active transporters, and the competition with the cells for the surface of the cell culture plates, thereby promoting cell detachment (Li et al., 2009, Robert et al., 2020). However, the treatment with the quercetin-loaded liposomes was the most effective in reducing the metabolic activity of the HCT-116 p53^+/+^ cell line.

In conclusion, colon cancer cells resulted significantly (*p* < 0.05) more sensitive to quercetin-loaded liposomes compared to free quercetin, meaning that quercetin-loaded liposomes could represent an efficient delivery system to be further explored.

## 4 Conclusions

HPH was found to be an effective method to obtain nano-sized liposomes for the delivery of quercetin. The process conducted at 150 MPa for a single pass was the most efficient for producing liposomes with the highest encapsulation efficiency and the lowest dimensions. Notably, the increase in process intensity up to a certain level reduced its performance, highlighting the pivotal role of process optimization to avoid the over-processing phenomenon.

Finally, the application of multiple analytical techniques allows a detailed characterization of liposome particle size and morphology. The widely applied batch mode DLS was proven to be a low-resolution technique for the characterization of these polydisperse systems. The quercetin-loaded nano-sized liposomes showed a good capacity to act as an antitumor agent in colon cancer cells in comparison to free quercetin and unloaded systems. Even if further studies are needed to demonstrate the behaviour of HPH-liposomes in food products and in the human body, these findings suggest that HPH can be proposed as a sustainable tool for the encapsulation and delivery of bioactive molecules for different purposes.

## CRediT authorship contribution statement

**Sofia Melchior:** Formal analysis, Investigation, Data curation, Methodology, Writing – original draft, Visualization, Writing – review & editing. **Marta Codrich:** Formal analysis, Investigation, Data curation, Writing – original draft, Visualization, Writing – review & editing. **Andrea Gorassini:** Formal analysis, Investigation, Data curation, Visualization, Writing – original draft, Visualization, Writing – review & editing. **Dora Mehn:** Formal analysis, Investigation, Methodology, Visualization, Writing – review & editing. **Jessica Ponti:** Formal analysis, Investigation, Writing – review & editing. **Giancarlo Verardo:** Formal analysis, Investigation, Data curation, Visualization, Writing – original draft, Visualization, Writing – review & editing. **Gianluca Tell:** Writing – original draft, Resources, Visualization, Writing – review & editing, Supervision. **Luigi Calzolai:** Visualization, Supervision, Resources, Writing – review & editing. **Sonia Calligaris:** Supervision, Visualization, Resources, Writing – original draft, Writing – review & editing.

## Declaration of Competing Interest

The authors declare that they have no known competing financial interests or personal relationships that could have appeared to influence the work reported in this paper.

## Data Availability

Data will be made available on request.
