# Supplementary figures and images for: Design and advanced characterization of quercetin-loaded nano-liposomes prepared by high-pressure homogenization
Source: Food Chem. 2023 Dec 1;428:136680. doi: 10.1016/j.foodchem.2023.136680 (PMC10410694; doi:10.1016/j.foodchem.2023.136680)

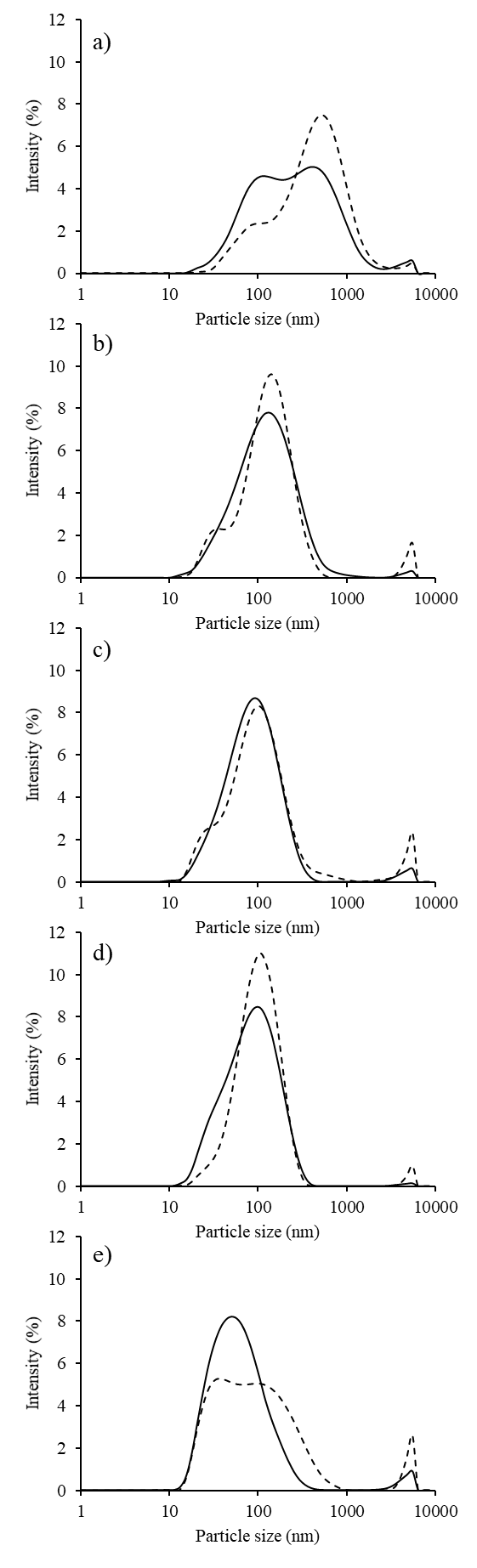


Figure S 1

Supplement: Supplementary data 1 [file mmc1.docx]
